# Impact of perceived ease of use, organizational support mechanism, and industry competitive pressure on physicians’ use of liver cancer screening technology in medical alliances

**DOI:** 10.3389/fpubh.2023.1174334

**Published:** 2023-08-03

**Authors:** Junhong Lu, Qingwen Deng, Yuehua Chen, Wenbin Liu

**Affiliations:** School of Health Management, Fujian Medical University, Fuzhou, China

**Keywords:** technology diffusion, physician, technology-organization-environment (TOE) framework, mindsponge theory, structural equation model (SEM), contrast-enhanced ultrasound (CEUS)

## Abstract

**Background:**

Liver cancer is one of the malignant tumors worldwide, while the prevention and control situation is grim at present, and the diffusion of its early screening technology still faces some challenges. This study aims to investigate the influencing mechanism of perceived ease of use, organizational support mechanism, and industry competitive pressure on hepatic early screening technologies use by physicians, so as to promote the wider use of corresponding technologies.

**Methods:**

Under the theoretical guidance of technology-organization-environment framework and mindsponge theory, this study took hepatic contrast-enhanced ultrasound as an example, and conducted a cross-sectional questionnaire by randomly selecting physicians from Fujian and Jiangxi provinces in China with a high and low incidence of liver cancer, respectively. Structural equation modeling was used to determine the correlation among perceived ease of use, organizational support mechanism, and industry competitive pressure, as well as their impact on the physicians’ behavior toward contrast-enhanced ultrasound use.

**Results:**

The hypothesis model fits well with the data (*χ*^2^/df = 1.863, GFI = 0.937, AGFI = 0.908, RMSEA = 0.054, NFI = 0.959, IFI = 0.980, CFI = 0.980). Under technology-organization-environment framework, the perceived ease of use (*β* = 0.171, *p* < 0.05), organizational support mechanism (*β* = 0.423, *p* < 0.01), industry competitive pressure (*β* = 0.159, *p* < 0.05) significantly influenced physicians’ use of hepatic contrast-enhanced ultrasound. Besides, perceived ease of use and organizational support mechanism (*β* = 0.216, *p* < 0.01), perceived ease of use and industry competitive pressure (*β* = 0.671, p < 0.01), organizational support mechanism and industry competitive pressure (*β* = 0.330, *p* < 0.01) were all associated significantly.

**Conclusion:**

From the lens of information processing (mindsponge theory) and technology-organization-environment framework, this study clarified the social and psychological influencing mechanism of perceived ease of use, organizational support mechanism, and industry competitive pressure on physicians’ use of hepatic contrast-enhanced ultrasound. The results will directly propose recommendations for expanding hepatic contrast-enhanced ultrasound utilization and indirectly promoting other appropriate and effective health technologies diffusion within the integrated health system.

## 1. Introduction

Liver cancer is one of the major global health challenges and is associated with a high mortality rate (1, 2), with approximately 2.96 million people currently infected with hepatitis B and 1 million deaths per year from HBV-related causes (including cirrhosis and liver cancer) (3). Symptoms of liver cancer are generally not obvious in the early stages, and once abdominal pain or palpation of an abdominal mass occurs, it is considered to be at the advanced stage. Even if treatment is given at this stage, health outcomes are not promising. Thus, it is of great importance to improve the early detection and treatment of liver cancer to get better the prognosis of patients. As a non-invasive and reproducible imaging technique (4), contrast-enhanced ultrasound (CEUS) has been proven to be effective and appropriate in diagnosing liver cancer in the subclinical stage of asymptomatic and subclinical symptoms (5, 6). And it has been recognized to be a cost-effective tool by various national and regional health institutions and can be widely utilized as a first-line imaging method for the cure and prognosis of liver cancer (7, 8). Although certain primary health institutions are qualified or already equipped with ultrasound equipment, CEUS has not been broadly applied in primary health institutions in China (9), resulting in a greatly weaken role in early cancer detection and diagnosis in primary health institutions (10). Therefore, to advance the early prevention and control of liver cancer in the whole population, it is urgent to examine the influencing mechanisms of the utilization of liver cancer screening technology to promote its clinical practice and application in the medical alliance.

Prior research on individual decision or technology utilization concentrates on biology, neurology, and psychology (11), and employs utility maximization to rationally assess the expected outcome, represented by prospect theory, mindsponge theory, theory of planned behavior, technology acceptance model, technology-organization-environment framework, and so on. For example, prospect theory explains the non-psychological factors that affect the choice behavior from the psychological and behavioral characteristics (12). Mindsponge theory, which holds that individuals evaluate their acceptance of new values (information) from the external environment based on cost-benefit judgments and trust assessment (13, 14), explicitly illustrates how and why individuals receive new values from an information processing lens. Additionally, technology acceptance model (TAM), incorporating theory of rational action and theory of planned behavior, argues that technology utilization is dependent on perceived ease of use and usefulness (15). Finally, the technology-organization-environment (TOE) framework integrates predictors at the technological, organizational, and environmental levels to provide a systematic horizon of barriers and enablers in technology utilization process (16, 17). The above theoretical framework has some explanatory power to interpret technology utilization or individual decision-making. While in-depth analysis revealed that TAM centers on the technology nature (18, 19); and prospect theory is mainly applied to economics, law, and politics (20), its application scope and explanatory power in the health field needed to be expanded. Whereas TOE framework is considered the most appropriate and general for understanding technology utilization (21), focusing on specific technologies through norms and adaptations that ultimately promote meaningful research.

At present, scholars base their research on the TOE framework, which has been proven in numerous empirical surveys for its wide-ranging utility and its ability to interpret and predict in complex contexts, most of which concentrated on technology utilization in different types of economic sectors, such as government agencies, enterprises (inter-organizational information systems, knowledge management systems, electronic data exchange) and specific industries (health, retail, manufacturing). Currently, under the guidance of the TOE framework, some research observes the influence factors of emerging high-tech (cloud computing) (22–24). Studies on technology adoption in Chinese enterprises have also identified environmental and organizational determinants involving customer authorization, competitive pressure, supplier or top management support, and technology maturity, expertise, and company size (25, 26). In recent years, mindsponge theory has been used to investigate the decision-making process of technological innovation and risk behavior, covering vaccine production and vaccination (27, 28), enterprise innovation capacity (29, 30), and suicide behavior (31, 32). Research has shown that mindsponge theory adapts to the surrounding environment by continuously absorbing and excluding information to ensure a more logical interpretation about the effects of environmental factors on behavior.

Therefore, this study will take hepatic CEUS technology as an example and use the TOE framework and Mindsponge Theory as guidance, and apply structural equation modeling (SEM) to comprehensively determine the influencing mechanism of perceived ease of use, organizational support mechanism, and industry competitive pressure on physicians’ CEUS use in medical alliances. This study will not only benefit to clarify the influencing mechanism of certain technology use, but also provide an effective method to survey the psychological process behind technology innovation or diffusion especially in the setting of integrated care systems. Overall, understanding the barriers to the diffusion of liver cancer screening technology will provide evidence and recommendations for the smooth adoption and utilization of technology, and further provide guidance for promoting the diffusion and utilization of other appropriate and effective health technologies in the integrated health services system.

## 2. Methods

### 2.1. The hypothesized model

**Figure 1:**
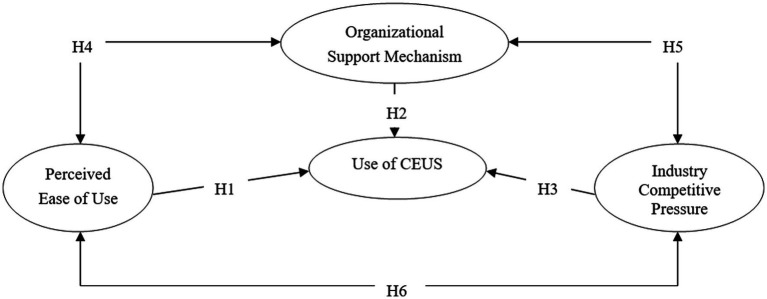
The theoretical model.

The technology-organization-environment framework is an organizational-level theory that illustrates the determinants of innovative technology processes that arise from technological context, organizational context, and environmental context. The broad applicability of the theory ensures that researchers can verify its application in specific contexts (33). Additionally, this study picked representative factors, namely PEOU, organizational support mechanism, and industry competitive pressure, at the technological level, organizational level and environmental level to construct models and validate their impact on technology utilization. Several empirical studies have confirmed that ease of use, organization support and external pressure are the essential predictive factors (21, 34). Therefore, it can be inferred that they positively influence CEUS utilization in medical alliances and correlate with each other. The proposed theoretical model is presented in Figure 1, and the above paths are represented by hypotheses 1 to 6:

*H1*: Physicians’ use of the hepatic CEUS technology is related to their PEOU.

*H2*: Physicians’ use of the hepatic CEUS technology is related to organizational support mechanism.

*H3*: Physicians’ use of the hepatic CEUS technology is related to industry competitive pressure.

*H4*: PEOU and organizational support mechanism associate with each other.

*H5*: Organizational support mechanism and industry competitive pressure associate with each other.

*H6*: PEOU and industry competitive pressure associate with each other.

### 2.2. Instrument

Guided by the proposed theoretical model, a literature-research and expert-discussed was designed a questionnaire, comprising three sections. The first section entailed demographic characteristics (e.g., gender, age, education, professional titles, administration position and years in practice). The second section was the probability of physicians using CEUS to diagnose liver cancer in the context of technological applicability. Estabrooks (35) and Beyer (36) divide the utilization structure into conceptual, instrumental and persuasive use, so the study measured the different levels of CEUS use by the probability of proficient use, actual prescription, and making recommendations to peers, where 0 = never, 1 = very low (0%–20%), 2 = low (20%–40%), 3 = medium (40%–60%), 4 = high (60%–80%), and 5 = very high (80%–100%). The third section included PEOU, organizational support mechanism and industry competitive pressure, consisting of 11 items measured on a five-point Likert scale with “strongly disagree,” “disagree,” “neutral,” “agree,” and “strongly agree” recorded as 1, 2, 3, 4, and 5. (1) PEOU is physicians’ perceived ease of use on CEUS measured by the ease of access to material, equipment and test results, and these items adapted from Bhattacherjee (37) and Hsiao et al. (38) designed scales with excellent reliability and validity. (2) Organizational support mechanism is human, material or other support provided by the health institutions to advance CEUS application, as measured by funding support, the installation of dedicated staff, and the establishment of information feedback channels, referencing Helfrich et al. (39) and Grover’s (40) representative scales. (3) Industry competitive pressure is perceived pressure from competitors, partners, and the healthcare industry to adopt CEUS, which in turn forces hospitals and physicians to learn and apply it. These items were referenced to scales with convincing reliability and validity (41, 42). References for the scale design are detailed in Supplementary file 2.

### 2.3. Sampling and data collection

During the period from February to August 2019, a cross-sectional survey was conducted in Fujian and Jiangxi Provinces using a multi-stage sampling method. Firstly, Fujian (32.18/100000) (43) and Jiangxi (23.80/100000) (44) provinces were randomly selected from the provinces with a high (Fujian, Jiangsu, Guangxi, Guangdong, etc.) and low (Jiangxi, Hubei, Shanxi, Hebei, etc.) incidence of liver cancer in China. Secondly, two medical alliances were randomly selected from each province and half of the health institutions in each medical alliance were surveyed to ensure an adequate study population. Thirdly, all physicians with CEUS knowledge and working in liver disease-related departments (hepatology, oncology, gastroenterology, infection, radiotherapy, interventional, ultrasound, etc.) were considered as the study population. This is because technology utilization is hierarchical and requires attention not only to the direct users, but also to the individuals who may be involved in the process of using CEUS, whose active behavior may drive the adoption and diffusion of CEUS. Ultimately, it was expected that 5–8 health institutions would be investigated in each selected medical alliance, for a total of 20–30 health institutions would be surveyed. Since an average of 10 to 20 physicians will be surveyed at each sampled institution, probably more than 200 physicians would participate in this study, fully meeting the basic requirement that the sample size be set at least 5 times the question (45).

This study was approved by the Ethics Committee of Fujian Medical University (No. 2017-17). Supported by the surveyed institutions, a highly trained volunteer was available to accompany each round for filling out the questionnaire, who explained the study purpose and data use in detail to ensure the participants understand what they needed to do and how to do it. These surveys were conducted anonymously to protect personal privacy, but the participants were encouraged to submit their contact information if they were interested in the results of the study.

### 2.4. Statistical analysis

Firstly, descriptive analyses were implemented to present the participants’ demographic characteristics and measurement scores. Secondly, the reliability and validity of the questionnaire were assessed via Cronbach’s *α* coefficient and factor analysis. Finally, SEM was carried out to verify the proposed hypotheses and accordingly determine the mechanism influencing physicians’ using the hepatic CEUS technology. Chi-square/df, GFI (goodness-of-fit index), AGFI (adjust the goodness-of-fit index), root-mean-square error of approximation (RMSEA), and CFI (comparative fit index) were used for checking the model fit. SPSS 21.0 and Amos 17.0 were applied to perform the data analysis. In all reported analyses, *p* < 0.05 was considered as statistically significant.

## 3. Results

### 3.1. Characteristics of the participants

Table 1 demonstrates the demographic characteristics of the participants. A total of 329 physicians responded to the questionnaires. After excluding 28 questionnaires for missing data or same responses to all items, there were 301 valid questionnaires with a valid response rate of 91.5%. Of the participants, most were under 45 years old and 193 (64.1%) were male. More than half of them (58.1%) had an undergraduate degree. The majority of the participants did not hold management positions, but more than half obtained intermediate or higher professional titles.

**Table 1:** Demographic characteristics of the participants.

Characteristic	*N*	Percent (%)
*Gender*		
Male	193	64.1
Female	108	35.9
*Age group (years)*		
<35	133	44.2
35–45	122	40.5
>45	46	15.3
*Education level*		
College and below	27	9.0
Undergraduate	175	58.1
Master’s degree or above	99	32.9
*Professional titles*		
Junior	104	34.5
Intermediate	123	40.9
Senior	74	24.6
*Administration position*		
Yes	57	18.9
No	244	81.1
*Years in practice*		
<5 years	72	23.9
5–10 years	95	31.6
11–20 years	91	30.2
21–30 years	35	11.6
30 years	8	2.7

### 3.2. Reliability and validity

Table 2 reports Cronbach’s α, composite reliability (CR), and average variance extracted (AVE) of all constructs. The Cronbach’s *α* of 4 dimensions and the entire questionnaire were all greater than the recommended 0.7 threshold (46), ranging from 0.847 to 0.930, indicating adequate internal consistency and good reliability. Besides, the factor loading values of all items in the four dimensions were greater than 0.5. What’s more, the CR values of the dimensions were all above 0.8 (the technological dimension is very close to 0.8) and AVE values of all constructs were above 0.5, which indicated an acceptable convergent validity.

**Table 2:** Results of confirmatory factor analysis.

Dimension	Item	Cronbach’s *α*	CR	AVE	Factor loading	*R* ^2^	*p*-value
Technology	PEOU1	0.851	0.799	0.609	0.703	0.494	
	PEOU2				0.748	0.560	<0.001
	PEOU3				0.865	0.749	<0.001
	PEOU4				0.796	0.634	<0.001
Organization	OSM1	0.934	0.967	0.833	0.847	0.717	
	OSM2				0.974	0.949	<0.001
	OSM3				0.912	0.832	<0.001
Environment	ICP1	0.887	0.861	0.672	0.745	0.555	
	ICP2				0.792	0.627	<0.001
	ICP3				0.881	0.776	<0.001
	ICP4				0.853	0.728	<0.001
Use	U1	0.930	0.961	0.819	0.854	0.729	
	U2				0.920	0.847	<0.001
	U3				0.939	0.881	<0.001

CR, composite reliability; AVE, average extracted variance.

### 3.3. Measurement scores of all predictors and use of CEUS

The specific measurement scores of each item under the three dimensions are shown in Table 3. Three predictors of PEOU, organizational support mechanism, and industry competitive pressure had a mean score of 4.12 [standard deviation (SD): 0.75], 2.66 (SD: 1.16), and 3.86 (SD: 0.88), respectively. The mean score of the use of CEUS was 1.93 with a standard deviation of 1.27.

**Table 3:** Measurement scores of participants.

Measurements	Mean	SD	Median	*N* (%) of scores > mean
Perceived ease of use	4.12	0.75	4.00	136 (45.18%)
PEOU1	4.25	0.82	4.00	139 (46.18%)
PEOU2	3.85	1.07	4.00	191 (63.46%)
PEOU3	4.12	0.91	4.00	125 (41.53%)
PEOU4	4.27	0.80	4.00	143 (47.51%)
Organizational support mechanism	2.66	1.16	3.00	178 (59.14%)
OSM1	2.53	1.23	3.00	168 (55.81%)
OSM2	2.69	1.26	3.00	182 (60.47%)
OSM3	2.75	1.25	3.00	188 (62.46%)
Industry competitive pressure	3.86	0.88	4.00	179 (59.47%)
ICP1	4.02	0.96	4.00	116 (38.54%)
ICP2	3.65	1.14	4.00	164 (55.49%)
ICP3	3.86	1.01	4.00	187 (62.13%)
ICP4	3.92	0.97	4.00	204 (67.77%)
Use of CEUS	1.93	1.27	1.00	110 (36.54%)
U1	1.80	1.26	1.00	106 (35.22%)
U2	2.06	1.43	1.00	101 (33.55%)
U3	1.92	1.38	1.00	110 (36.54%)

### 3.4. Structural equation models

All model fit indices were within the acceptable range: p-value <0.001, *χ*^2^/df = 1.863 (<5), GFI = 0.937 (>0.9), AGFI = 0.908 (>0.9), RMSEA = 0.054 (<0.08), NFI = 0.959 (≥0.90), CFI = 0.980 (>0.9), which indicated that the research model has fit the data well.

**Figure 2:**
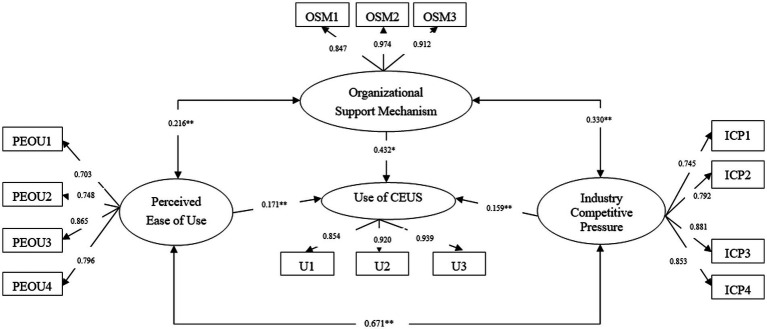
Model of utilization behavior of CEUS among 301 physicians.

After the measurement model was confirmed, we used SEM to verify the proposed hypotheses. The structural model with standardized estimates is presented in Figure 2. There was a significant correlation among the PEOU, organizational support mechanism, and industry competitive pressure, while no indirect effect between these three factors on CEUS use. The correlation coefficient between PEOU and organizational support mechanism is 0.216 (*p* < 0.05); as well as the correlation coefficient between PEOU and industry competitive pressure is 0.671 (*p* < 0.05); and the correlation coefficient between organizational support mechanism and industry competitive pressure is 0.330 (*p* < 0.05). Furthermore, regarding the influencing mechanism of physicians’ CEUS use in the medical alliance, the model showed that at the technological level, the PEOU predicted the behavior of physicians’ using hepatic CEUS (*β* = 0.171, *p* < 0.05). At the organizational level, the organizational support mechanism was associated with the behavior of physicians’ using hepatic CEUS (*β* = 0.432, *p* < 0.001). At the environmental level, the industry competitive pressure was linked to the behavior of physicians’ using hepatic CEUS (*β* = 0.159, *p* < 0.05). In particular, the organizational support mechanism had the strongest impact on the use of hepatic CEUS technology (0.432), followed by the PEOU (0.171) and the industry competitive pressure (0.159).

## 4. Discussion

Under the current context of the severe situation of liver cancer prevention and control, as well as the uneven diffusion and utilization of liver cancer screening technology in the medical alliance, determining the potential influencing factors at different levels (e.g., technological, organizational, environmental) and investigating their influencing mechanism on liver cancer screening technology adoption are of vital importance. Based on the TOE framework and mindsponge theory, a hypothesized model was established to investigate the potential factors, namely PEOU, organizational support mechanism, and industry competitive pressure on physicians’ CEUS use were examined detailed from the perspective of information processing. These findings will not only provide new perspectives to promote the vertical integration and optimization of other high-quality health resources within medical alliances, but also contribute insights into the psychological process of new technologies adoption in the medical alliances.

Since the relationship among technological context, organizational context, and environmental context has been confirmed in various studies on technology adoption, such as artificial intelligence health (47) and mobile health system (48, 49), the empirical result of this study also support the point. The qualitative study of commercial health insurance drivers proved that three factors influence each other to achieve the performance configuration of insurance payment (50). It can be further inferred that the factors at the technological level, organizational level and environmental level may interact and ultimately influence technology utilization in medical alliances. Thus, the importance should be stressed on not only tailoring strategies at specific levels, but also coordinating the effects of multiple measures from different levels.

Consistent with prior findings demonstrating that PEOU will facilitate the utilization and diffusion of mobile health technologies (51) and electronic personal health record (52, 53), this study also indicated a positive correlation between PEOU and CEUS use. More specifically, physicians will proactively accept and use CEUS, if they perceived it does not require additional effort and time to master it, yet effectively improves liver cancer diagnosis sensitivity and reduce the death risk. Moreover, this echoes the information processing mechanism of mindsponge theory. In the study, the new value is physicians’ beliefs about CEUS use, they will thoroughly evaluate the potential costs and benefits through multiple filtering systems. It is not until clinical staff realized that new technology has more advantages, such as increased diagnostic rate and reduced disease burden, that they are inclined to integrate new insights with original values to apply CEUS into clinical practice when conditions are appropriate (29, 54). Therefore, in addition to focusing on the ease of use and diagnostic advantages, personnel need to be aware of the constructive feedback or positive impact that innovative technologies may have, ultimately fueling its diffusion.

Additionally, this study showed that organizational support mechanism also played an important role in CEUS utilization, similar to previous research that the lack of support from the main stakeholders may halt the wider implementation of the new health technology (55). Moreover, top management support means providing multiple channels of communication and feedback, financial and technological support, and the long-term strategic vision and commitment to create a positive environment suitable for change, which provides a priority pass to introduce new technologies and enhance the sense of belonging and connection between physicians and hospitals (32). Therefore, in the information filtering process, physicians relax their perception about the cost–benefit of new technologies out of trust in the diverse support provided by hospitals (14), and their positive interaction with each other clear away invisible barriers to technology use, which enhances the presence and accessibility of new technologies in the mindset.

As reported in previous research on the TOE framework, external industry competitive pressure was one of the key dimensions of the environmental context (56). In line with prior studies (51, 56), this study demonstrated that industry competitive pressure strongly affects CEUS utilization. Specifically, for the member institutions within the context of the medical alliance, such pressure is not only from the competing hospitals outside the medical alliance but also from the internal cooperative institutions (57). Especially if certain technology was capable of greatly improving the diagnostic efficacy or reducing the operation cost, managers will have few choices but to take swift actions to expand its use. This is also in line with mindsponge theory, where an individual’s mindset is constantly updated and strengthened to adapt to the changes in the external environment (13, 54). As industry competitors or partners in the supply chain are pushing for technology use, physicians or hospitals has to accept changes in the external environment to protect their leading status or revenue, which in turn increases the perceived value of relevant technological information and makes acceptance easier.

According to the understanding of the behavioral mechanisms of physicians’ CEUS use, several interventions can be highlighted to further promote the adoption and diffusion of health technologies. For hospital administrators, emphasis should be placed on formulating explicit measures and coordinating multiple levels, namely technological, organizational, and environmental contexts. First, in addition to emphasizing the perceived ease of use, users should also be made aware of the potential benefits of using technology (58). Second, in addition to providing specific support (e.g., funding, equipment, systems), technological seminars and training sessions can be strengthened the links between physicians and hospitals and ensure smooth implementation (59). Finally, even though the intense industry competition, it is recommended that hospital administrators adequately rely on policy support to introduce new technologies after taking stock of the situation (15), which helps turn industry competitive pressure into a driver for technology diffusion.

This study has some strengths. Firstly, guided by the TOE framework, it comprehensively took the predictors from the technological, organizational, and environmental context into account, rather than merely focusing on a single level. Secondly, this study further interpreted the social and psychological process of CEUS use in a more complex context through an information processing mechanism (mindsponge theory), which will conducive to ensure the smooth implementation of certain liver cancer screening technologies. And also had some room for improvement. First of all, we cannot rule out the possibility that other factors may not be covered in the comprehensive influencing mechanism under the TOE framework. Follow-up studies are strongly recommended to include more intervention-relevant factors. Secondly, since participants were from only two sample areas and taking CEUS as an example may affect generalizability, future research could expand the range of sample sources.

## 5. Conclusion

Under the guidance of the TOE framework and mindsponge theory, this study used structural equation modeling to conduct empirical research and found that organizational support mechanism have the greatest relative impact on physicians’ use of liver cancer screening technology, followed by PEOU and industry competitive pressure. In addition, the study also revealed a close correlation between these three factors. Therefore, to promote the diffusion and utilization of corresponding health technologies, it is recommended to pay more attention to the performance advantages of technology, establish multiple support mechanisms such as funds and departments, and cooperate with other member institutions of the medical alliance to transform pressure of industry competition into driving forces of regarding technology diffusion.

## Data availability statement

The raw data supporting the conclusions of this article will be made available by the authors, without undue reservation.

## Ethics statement

The studies involving human participants were reviewed and approved by the Ethics Committee of Fujian Medical University (No. 2017-17). The patients/participants provided their written informed consent to participate in this study.

## Author contributions

WL designed and conducted the project, contributed to grasp the subject, and revised the manuscript. JL carried out the data analysis and drafted the manuscript. WL and QD developed the questionnaire. YC participated in the manuscript revision. All authors contributed to the article and approved the submitted version.

## Funding

This research was supported by the National Natural Science Foundation of China (grant numbers: 72274035 and 71704026) and the Distinguished Young Scientific Research Talents Plan in Universities of Fujian Province (grant number: 2018B030). The funders had no involvement in study design, data collection, statistical analysis, and manuscript writing.

## Conflict of interest

The authors declare that the research was conducted in the absence of any commercial or financial relationships that could be construed as a potential conflict of interest.

## Publisher’s note

All claims expressed in this article are solely those of the authors and do not necessarily represent those of their affiliated organizations, or those of the publisher, the editors and the reviewers. Any product that may be evaluated in this article, or claim that may be made by its manufacturer, is not guaranteed or endorsed by the publisher.

## Supplementary material

The Supplementary material for this article can be found online at: https://www.frontiersin.org/articles/10.3389/fpubh.2023.1174334/full#supplementary-material
