# Supplementary material for: Impact of perceived ease of use, organizational support mechanism, and industry competitive pressure on physicians’ use of liver cancer screening technology in medical alliances
Source: Front Public Health. 2023 Aug 3;11:1174334. doi: 10.3389/fpubh.2023.1174334 (PMC10434768; doi:10.3389/fpubh.2023.1174334)
Supplement: Supplementary file 1 [file Data_Sheet_1.docx]

Impact of perceived ease of use, organizational support mechanism, and industry competitive pressure on physicians’ use of liver cancer screening technology in medical alliance

Junhong Lu ^1^, Qingwen Deng ^1^, Yuehua Chen^1^, Wenbin Liu^1*^

*** Correspondence:** Wenbin Liu: [wenbinliu126@126.com](mailto:wenbinliu126@126.com)

**The questionnaire on the factors of the intention to use liver cancer screening among physicians**

**Part 1. Personal Information Card**

Notes. The following questions are some basic information about you, please circle the number or fill in the blank that best matches your real situation.

1. Gender:

A. Male B. Female

2. Age: ________

3. Educational level:

A. Junior college or below B. Bachelor C. Master D. Doctor

4. Professional title:

A. Junior B. Intermediate C. Senior

5. Do you have administration position:

A. Yes B. No

6. Years in practice:

A. <5 years B. 5~10 years C. 11~15years D. 16~20 years E. >20 years

**Part 2. Behavior to use contrast-enhanced ultrasound (CEUS)**

Notes. There are 6 numbers (0, 1, 2, 3, 4, 5) on the right side of each item, where “0” means “never”, “1” means=“Very low (0-20%]”,“2”means “Low (20%-40%]”, “3” means “Medium (40%-60%]”, “4” means “High (60%-80%]”, and “5” means “Very high (80%-100%]” . Please tick or circle the number that best fits your real feelings on the item.

| In the past year, the probability that I use CEUS on all working days. | 0 | 1 | 2 | 3 | 4 | 5 |
| --- | --- | --- | --- | --- | --- | --- |
| In the past year, the probability that I skillfully combine the CEUS results to make clinical diagnosis. | 0 | 1 | 2 | 3 | 4 | 5 |
| In the past year, the probability that I recommended further using CEUS to my peers. | 0 | 1 | 2 | 3 | 4 | 5 |

**Part 3. The scale of Theory of Planned Behaviour**

Notes. There are 5 numbers (1, 2, 3, 4,5) on the right side of each item, where “1” means “Strongly disagree”, “2” means “Disagree”, “3” means “Neutral”, “4” means “Agree”, and “5” means “Strongly agree”. Please tick or circle the number that best fits your real feelings on the item.

| ***Perceived ease of use*** | | | | | |
| --- | --- | --- | --- | --- | --- |
| We can easily obtain the materials and instruments needed for CEUS test. | 1 | 2 | 3 | 4 | 5 |
| We can easily obtain the equipment and reagents necessary for CEUS. | 1 | 2 | 3 | 4 | 5 |
| We can get the result of CEUS test in a short time after detection. | 1 | 2 | 3 | 4 | 5 |
| We can be provided with assistance in clinical diagnosis by the result of CEUS test. | 1 | 2 | 3 | 4 | 5 |
| ***Organizational support mechanism*** | | | | | |
| Hospital provided funding support for the introduction of CEUS technology to carry out related clinical services. | 1 | 2 | 3 | 4 | 5 |
| Hospital have designated the department or personnel responsible for technology introduction and application in the hospital. | 1 | 2 | 3 | 4 | 5 |
| Information communication channels have been established by the hospital for timely feedback of problems. | 1 | 2 | 3 | 4 | 5 |
| ***Industry competitive pressure*** | | | | | |
| CEUS has been widely used for liver cancer screening in the medical industry. | 1 | 2 | 3 | 4 | 5 |
| Many surrounding hospitals are using CEUS for liver cancer screening. | 1 | 2 | 3 | 4 | 5 |
| Our business partners recommend CEUS for liver cancer screening. | 1 | 2 | 3 | 4 | 5 |
| The application of CEUS in liver cancer screening. has become routinized. | 1 | 2 | 3 | 4 | 5 |
