# Supplementary material for: Impact of perceived ease of use, organizational support mechanism, and industry competitive pressure on physicians’ use of liver cancer screening technology in medical alliances
Source: Front Public Health. 2023 Aug 3;11:1174334. doi: 10.3389/fpubh.2023.1174334 (PMC10434768; doi:10.3389/fpubh.2023.1174334)
Supplement: Supplementary file 2 [file Data_Sheet_2.docx]

Impact of perceived ease of use, organizational support mechanisms, and industry competition pressure on physicians’ use of liver cancer screening technology in medical alliance

Junhong Lu ^1^, Qingwen Deng ^1^, Yuehua Chen^1^, Wenbin Liu^1*^

*** Correspondence:** Wenbin Liu: [wenbinliu126@126.com](mailto:wenbinliu126@126.com)

**References for questionnaire design**

| **Variables** | **Items** | **References** |
| --- | --- | --- |
| **Technology use** | In the past year, the probability that I use CEUS on all working days | Estabrooks (34),  Beyer JM (35) |
|  | In the past year, the probability that I skillfully combine the CEUS results to make clinical diagnosis |  |
|  | In the past year, the probability that I recommended further using CEUS to my peers |  |
| **Perceived ease of use** | We can easily obtain the materials and instruments needed for CEUS test | Bhattacherjee (36),  Hsiao (37) |
|  | We can easily obtain the equipment and reagents necessary for CEUS |  |
|  | We can get the result of CEUS test in a short time after detection |  |
|  | We can be provided with assistance in clinical diagnosis by the result of CEUS test |  |
| **Organizational support mechanism** | Hospital provided funding support for the introduction of CEUS technology to carry out related clinical services | Helfrich (38),  Grover (39) |
|  | Hospital have designated the department or personnel responsible for technology introduction and application in the hospital |  |
|  | Information communication channels have been established by the hospital for timely feedback on problems |  |
| **Industry competitive pressure** | CEUS has been widely used for liver cancer screening in the medical industry | Premkumar (40),  Sheu (41) |
|  | Many surrounding hospitals are using CEUS for liver cancer screening |  |
|  | The application of CEUS in liver cancer screening. has become routinized |  |
